# UV-Vis quantification of the iron content in iteratively steam and HCl purified single-walled carbon nanotubes

**DOI:** 10.1371/journal.pone.0303359

**Published:** 2024-05-10

**Authors:** Markus Martincic, Gerard Tobías-Rossell

**Affiliations:** Institut de Ciència de Materiales de Barcelona (ICMAB-CSIC), Campus de la UAB, Bellaterra, Barcelona, Spain; VIT University, INDIA

## Abstract

As-produced carbon nanotubes contain impurities which can dominate the properties of the material and are thus undesired. Herein we present a multi-step purification treatment that combines the use of steam and hydrochloric acid in an iterative manner. This allows the reduction of the iron content down to 0.2 wt. % in samples of single-walled carbon nanotubes (SWCNTs). Remarkably, Raman spectroscopy analysis reveals that this purification strategy does not introduce structural defects into the SWCNTs’ backbone. To complete the study, we also report on a simplified approach for the quantitative assessment of iron using UV-Vis spectroscopy. The amount of metal in SWCNTs is assessed by dissolving in HCl the residue obtained after the complete combustion of the sample. This leads to the creation of hexaaquairon(III) chloride which allows the determination of the amount of iron, from the catalyst, by UV-Vis spectroscopy. The main advantage of the proposed strategy is that it does not require the use of additional complexing agents.

## Introduction

Nanotechnology offers new strategies to improve or even overcome some of the limitations of traditional materials [1–8]. Among the different types of nanomaterials, carbon nanotubes (CNTs) can be employed in a wide variety of applications that range from nanoelectronics, sensors, catalysis, composite materials and cancer therapy to name some [9–18]. The synthesis of CNTs results in samples that also contain both carbonaceous and non-carbonaceous impurities. These are typically amorphous carbon, fullerenes, graphitic onions, catalytic nanoparticles and catalytic particles. Furthermore, the catalytic particles could be coated by graphitic shells that protects them from direct dissolution. The presence of metal catalyst might not only induce toxicity [19,20] but, along with graphitic particles, can also affect and even dominate the electrochemical response of the sample [21–24]. High purity carbon nanotubes are indeed needed, for instance, for their application in the biomedical field to avoid undesired side effects [25–28]. The removal of impurities from the as-produced material is necessary for the application of carbon nanotubes in different fields, and various methods for this purpose have already been proposed [29–36]. The most widely employed strategies involve the use of strong oxidizing acids, such as nitric acid, which can induce structural damage to the nanotube walls. Other strategies for the purification of carbon nanotubes include their treatment in oxidizing atmospheres such as air or oxygen, hydrogen peroxide, microwave heating, ultrasounds, electro-purification and magnetic separation to name some [37–40]. High temperature annealing has proven to be efficient in the removal of metal catalysts from multi-walled CNT samples [41], but it can pose a problem when dealing with single-walled CNTs because (SWCNTs), at high temperature, coalescence of SWCNTs has been observed forming multi-walled CNTs [42,43]. There is not a best strategy to purify CNTs, because it largely depends on the targeted application. For instance, the presence of functional groups introduced by nitric acid treatment, increases the dispersability and processability of the material and cross-links between the individual carbon nanotubes [44]. This can be an advantage or disadvantage depending on the final application. Acid treated CNTs are of interest for their further derivatization with functional groups, doping, and as anchoring points for the in-situ growth of inorganic nanoparticles [45–48]. Recently, Tanaka *et al*. reported on a damage-less dry-purification of carbon nanotubes using FeCl_3_ vapor [49]. A low degree of structural defects is for instance desired for nanoelectronic devices and to prevent the undesired leakage of encapsulated materials when dealing with filled carbon nanotubes. We have placed our attention on the use of steam to efficiently eliminate impurities from carbon nanotubes without compromising their structural integrity and without introducing functional groups. Steam provides a gentle oxidizing atmosphere that effectively eliminates amorphous carbon and also graphitic particles, which might in turn contain metal catalysts in their interior. Following the steam purification, these metal particles become exposed (free from their protective graphitic coating) and can be readily dissolved in HCl [50]. Steam treatment has also been employed for the purification of highly aligned arrays of carbon nanotubes. A significant improvement in both quality, with respect to defect density, and in crystallinity, was observed resulting in an increased resistance to oxidation [51]. These super resilient carbon nanotubes were shown to withstand temperatures above 900°C under ambient conditions. The use of steam also results in the end opening of carbon nanotubes [52] thus allowing their posterior filling with a wide variety of payloads [53–55]. The carbon nanotube ends can then be sealed/closed thus allowing to remove the compounds that have not been encapsulated [56–59]. The confinement of materials inside the cavities of carbon nanotubes not only allows the formation of unprecedented structures but also expands their range of application [60–64]. Due to its gentle oxidizing properties, employing steam reduces the likelihood of introducing functional moieties or structural defects on the CNT structure [65].

Various techniques are employed to determine the amount of catalyst present in carbon nanotube samples, being inductively coupled plasma (ICP), thermogravimetric analysis (TGA), and SQUID the most frequently used. Agustina *et al*. reported on a colorimetric assay for quantifying iron in carbon nanotube samples [66]. In their study, the authors compared different acids for the complete digestion of the sample. Once digested, 1,10-phenanthroline was employed to form a red-orange complex with iron which could then be quantified. Herein, we propose a simplified approach that does not require the addition of a complexing agent to determine the amount of catalyst by ultraviolet-visible spectroscopy. The proposed strategy requires the use of a minimal amount of sample. For instance, the remaining residue after thermogravimetric analysis can be employed for its subsequent characterization by UV-Vis spectroscopy. UV-Vis is widely available in most laboratories and provides reliable data with a fast acquisition time. We also report for the first time a multi-step purification approach consisting on an iterative steam-HCl treatment that can effectively reduce the catalyst content in the samples.

## Materials and methods

Single-walled carbon nanotubes, *ca*. 200 mg, synthesized by chemical vapor deposition (CVD) (Elicarb, Thomas Swan Ltd.) were ground with an agate mortar and pestle. The ground SWCNTs were then introduced into a 4 cm silica tube, which was used as sample holder, and placed in the middle of a furnace. Argon was bubbled through hot water and the resulting argon/steam gas was introduced into the furnace and allowed to react with the carbon nanotubes at 900°C during for 4 h to eliminate amorphous carbon and graphitic particles. To dissolve the now exposed metal catalyst, the sample was treated with 200 mL of 6 M HCl (Panreac) at 110°C overnight. The sample was then washed several times with purified water until a neutral pH was achieved (by monitoring the filtrate) and subsequently dried at 80°C. Several purification steps were performed by repeating the argon/steam treatment followed by an HCl wash in an iterative process. To evaluate the effect of fractioning the steam treatment, a set of experiments was designed. In all of them the total amount of steam was kept at 6 hours. Namely, an iterative steam-HCl process was repeated six times (using 1 h steam), three times (using 3h steam), two times (using 3 h steam) and also a single time (6 h steam). The latter was included as a control. In all the cases an HCl wash was performed after each steam treatment to dissolve those metal particles that were exposed (after oxidation of the graphitic shells by steam).

Carbon nanotubes (CNTs), *ca*. 5 mg, were subjected to thermogravimetric analysis (TGA) using a STA 449 F1 Jupiter (NETZCH) under flowing air. TGA was performed by heating up to 900°C at 10°C min^-1^ with a gas flow rate of 25 mL min^-1^. The solid remaining after TGA contains oxidized iron, from the catalyst particles. This residue was recovered, dissolved in conc. HCl (50 mL), and employed for UV-Vis analysis to quantify the metal content (Cary 5.0, using 1 cm quartz cells).

Raman spectra was acquired between 100 and 2000 cm^-1^ with a wavelength of 632 nm (20 mW He-Ne laser; Lab Ram HR 800 Jobin Yvon). For Raman measurements, samples were sonicated in isopropyl alcohol (Panreac). Few drops were then deposited on a glass substrate and the solvent was slowly evaporated at *ca*. 85°C.

SQUID measurements were conducted in a Quantum design MPMS XL-7T instrument at 10 K using an external DC field. A magnetic field range of -50,000 to +50,000 Oe was employed. For these measurements, SWCNTs (*ca*. 6 mg per sample) were placed inside gelatin capsules. Glass wool was employed to prevent any sample movement.

Scanning electron microscopy was performed in a QUANTA FEI 200 FEG-ESEM equipped with an energy-dispersive X-ray spectroscopy (EDX) detector. Sample for analysis was deposited on top of a carbon tape.

To quantitatively determine the amount of iron using UV-Vis analysis, a calibration curve was generated by measuring five different concentrations of iron(III) oxide, dissolved in conc. hydrochloric acid, within the range of 0.2 to 1 μmol/dm^3^. To assess the amount of Fe in the SWCNTs after each treatment, the residue obtained after TGA was initially sonicated in few mL of conc. HCl. Subsequently, the volume was raised to 25 mL using volumetric flasks. The absorbance at 243 nm was fitted to the previously prepared calibration curve to determine the concentration of Fe in the different samples.

## Results and discussion

In this work we have employed a two-step purification strategy for SWCNTs that consists on an initial thermal annealing of CNTs under argon, in the presence of steam (H_2_O), followed by an HCl treatment. Steam has been reported to open the ends of CNTs, and to remove the most reactive (defective) CNTs, amorphous carbon and graphitic particles. The graphitic shells might in turn be coating catalytic NPs. After the steam treatment, the now exposed metal nanoparticles can be easily dissolved by HCl, the second step of the purification process. In fact, the acidic solution turns yellow due to the dissolution of the iron particles. The dissolved iron can subsequently be eliminated through filtration, and the SWCNTs can be recovered as a solid powder on top of the filter membrane.

**Fig 1:**
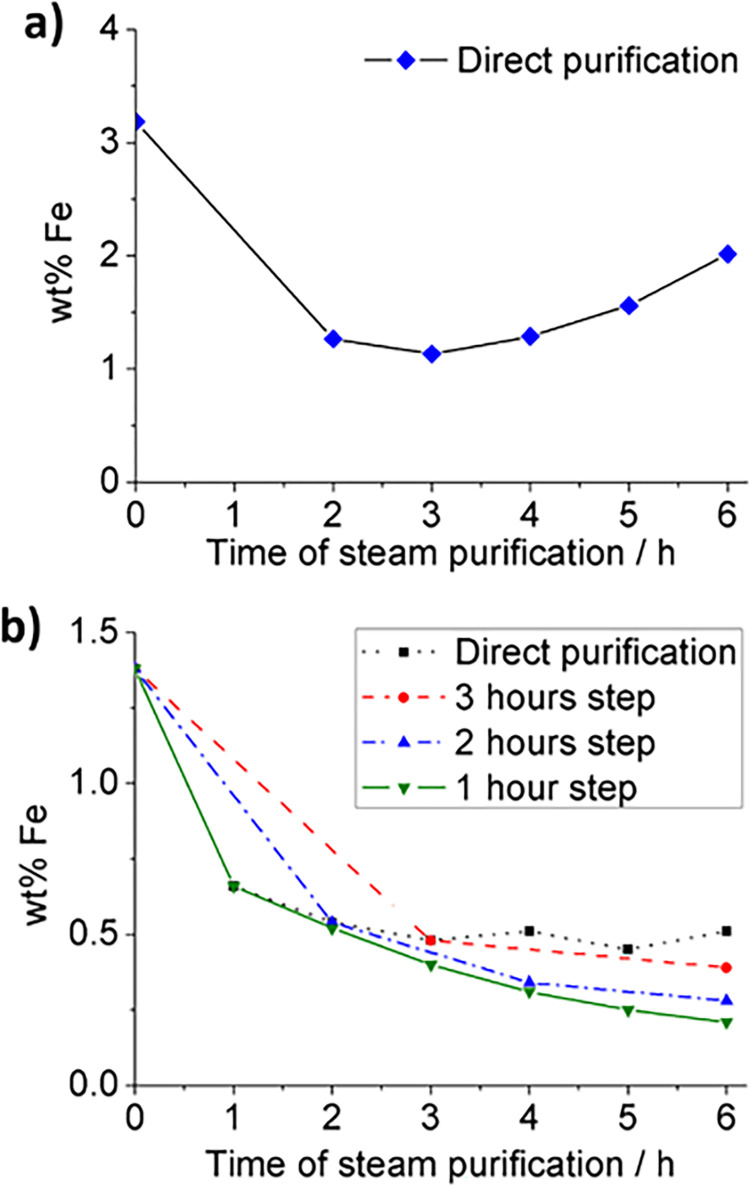
Iron content in as-received and purified SWCNTs. a) Amount of iron in samples of SWCNTs, which underwent a single steam treatment, “direct purification”, as assessed by TGA; all values were recorded after treating the sample with HCl. Both as-received SWCNTs (0 h) and steam (2 h, 3 h, 4 h, 5 h, 6 h) and HCl purified SWCNTs are included. b) Amount of iron determined by SQUID for both as-received SWCNTs (0 h) and iteratively purified SWCNTs. In this case, the steam treatment was conducted either one time for a given duration (“direct purification”) or in an iterative manner (1, 2, or 3 h steps), with a total steam treatment of 6 h. It is worth noting that two distinct batches of SWCNTs were used for the studies reported in (a) and (b).

Initially, we treated as-received SWCNTs for different periods of time with steam, followed by HCl. We will refer to this set of samples as “direct purification”. As it can be seen in Fig 1A, the application of steam on SWCNTs for a given time, with a subsequent HCl wash, yields samples with varying levels of inorganic residue. This is attributed to the presence of iron in the samples, as previously determined in Elicarb^®^ SWCNTs by X-ray photoelectron spectroscopy (XPS) analysis [50]. Initially, a decrease is observed up to three hours, followed by the subsequent apparent increase in the metal content. The minimum amount of iron (catalyst) was obtained after 3 h (1.1 wt% Fe) for this single-step steam treatment as determined by TGA (Fig 1A).

Iron in samples of SWCNTs can be quantitatively determined from the amount of residue present after TGA, using stoichiometric conversion. Iron undergoes oxidation to iron(III) oxide during the analysis in the presence of flowing air. As mentioned, after achieving the minimum metal content, an apparent rise of iron can be observed as the time of steam treatment increases. This phenomenon has been previously reported [50,52], and has been attributed to the fact that steam is more effective in oxidizing other carbonaceous species rather than the graphitic shells covering metal particles. TGA does not discern catalytic impurities from other inorganic compounds that can be present in the sample. Therefore, SQUID was next used to measure the amount of magnetic species, iron in the present study, in the samples. SQUID is considered an ultrasensitive technique that allows the quantitative determination of magnetic compounds in CNTs. The obtained data is presented in Fig 1B (solid black squares). In agreement with TGA, the obtained SQUID data also shows a decrease in the iron content after the purification treatment (steam + HCl) with respect to as-received SWCNTs. Noteworthy, the iron content remains practically constant, after 2 h of steam, within experimental error. Thus, the observed continuous increase of TGA residue, inorganic component of the sample, at 4 h, 5 h and 6 h might be due to the existence of additional inorganic compounds used in either the production of CNTs or their purification. Previous studies have identified the presence of alumina and silica in CNT samples produced through chemical vapor deposition [52,66].

In order to further reduce the iron content in SWCNT samples, the 6 h steam treatment was fractioned in two treatments of 3 h, with an HCl wash after each of them. Data shown in Fig 1B, indicated as “3-hour step” and denoted by solid red dots. A more significant reduction in catalyst content was observed with respect to the direct 6 h steam purification, even when the total treatment time was the same. This suggests a more efficient elimination of catalytic particles in the sample through the fragmentation of the purification process. Encouraged by this result, the steam treatment was further divided. The total exposure time of SWCNT samples to steam remained constant at 6 h, but the samples were collected either (i) every 1 h, from the steam purification system, (green solid triangles down-facing, Fig 1B) or (ii) every 2 h (blue solid triangles up-facing, Fig 1B). After each individual steam treatment, samples underwent an HCl wash. The amount of catalyst, in this case iron, was then quantified using SQUID. Notably, the lowest amount of Fe in the SWCNT samples was achieved by increasing the amount of iterations. This observation becomes most evident at the endpoints, in the samples that had received a total steam treatment of six hours (6-hour points in Fig 1B). The amount of catalyst could be reduced by approximately 40%, going from *ca*. 0.5 wt. % of iron with a single 6 h treatment to *ca*. 0.2 wt. % of iron by performing six independent steam treatments of 1 h. These results provide evidence of the enhanced effectiveness of the multi-step treatment approach compared to the one-step “direct purification”. The more iterations of steam + HCl are performed, using shorter steam treatment times, the lower becomes the amount of Fe in the samples. The reason behind this observation would need further investigation, but it could be due to a higher accessibility of steam to the whole SWCNT sample in the multi-step processing, because the material gets mixed during the acid treatment. Towards this end it would be interesting to perform the steam purification in a rotary tube furnace. Another possibility would be that a certain degree of reaction occurs between steam and SWCNT samples during heating and cooling of the furnace. This would result in slightly longer exposure times of the sample with steam when the purification is fractioned.

Next, we explored the use of UV-Vis spectroscopy for the quantitative assessment of the iron content in the prepared samples, as an alternative to SQUID and TGA, which are the most stablished techniques for the determination of catalyst in CNTs. Taking into account that hexaaquairon(III) chloride is readily formed when iron is dissolved in HCl, and that this iron complex can be directly quantified by UV-Vis spectroscopy, we investigated the use of UV-Vis for the quantitative determination of the amount of Fe present in SWCNT samples without the need of using additional complexing agents.

**Fig 2:**
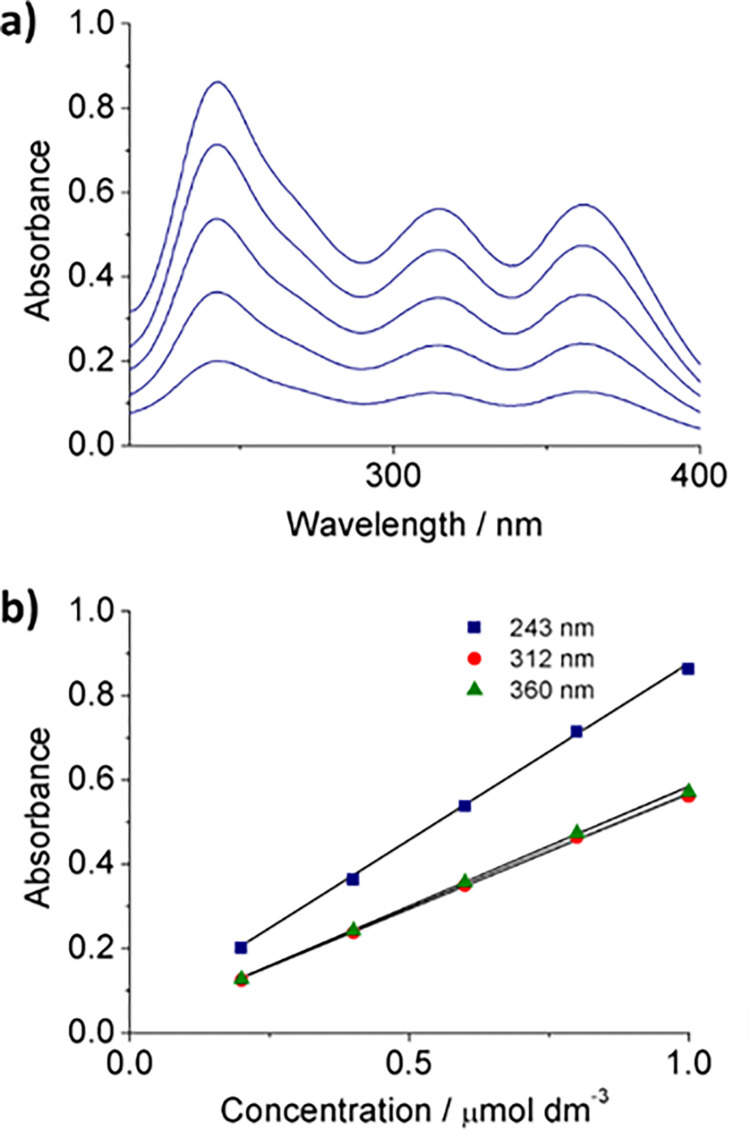
UV-Vis calibration curves. a) UV-Vis spectra obtained for the Fe_2_O_3_ standards and b) the resulting calibration curves using the recorded intensities of the three main peaks.

Initially UV-Vis calibration curves were prepared through the dissolution of known amounts of Fe_2_O_3_ in HCl. Fig 2 illustrates the recorded spectra alongside their respective calibration curves. Notably, three distinct peaks are evident. The peak at 243 nm, corresponding to the hexaaquairon(III), provides a more pronounced sensitivity:

  y_243_ = 0.0836 x + 0.0336; R^2^ = 0.9993

than the peaks at 312 nm, and 360 nm:

  y_312_ = 0.0549 x + 0.0182; R^2^ = 0.9992

  y_360_ = 0.056 x + 0.0186; R^2^ = 0.9990

Therefore, this peak (243 nm) was employed to determine the amount of Fe in the samples.

**Fig 3:**
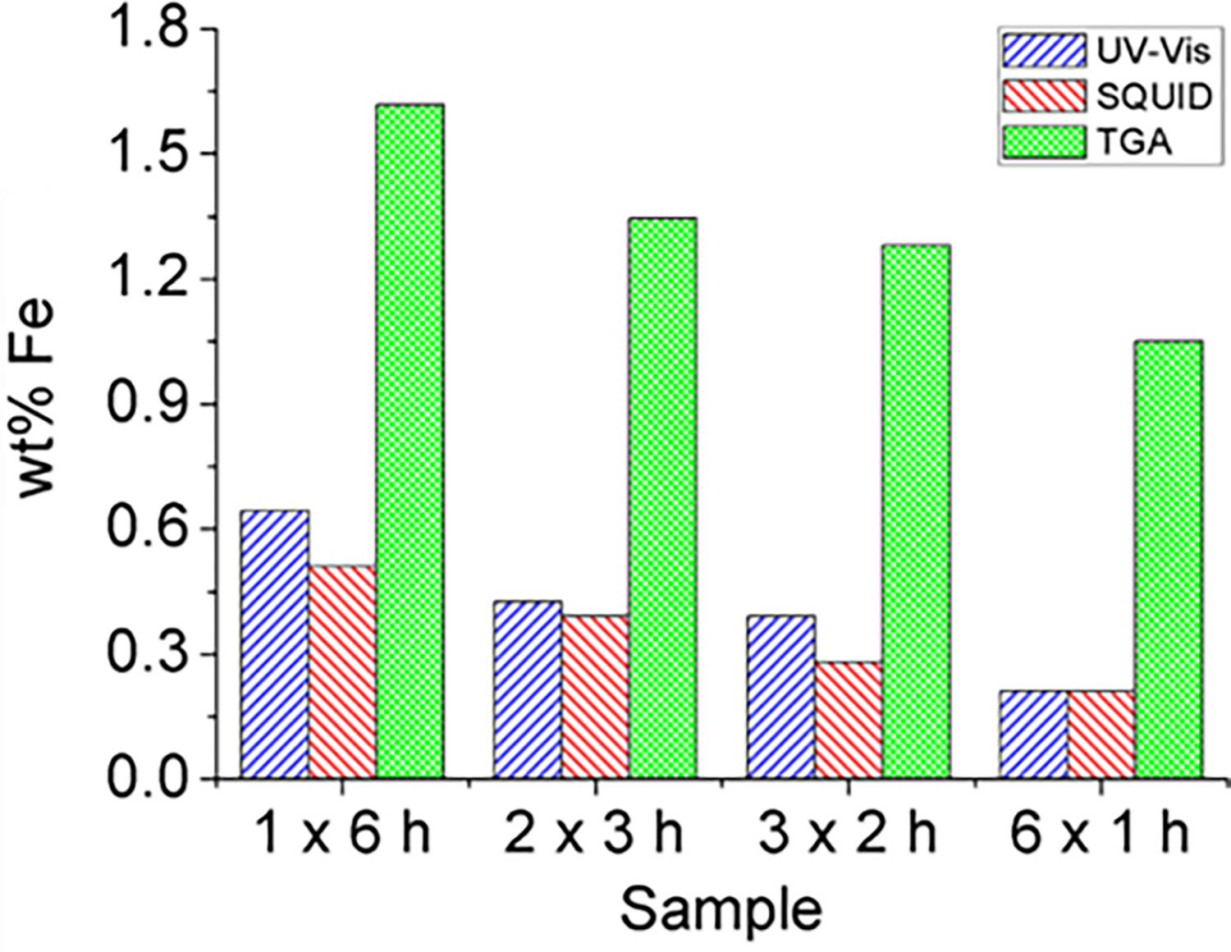
Quantitative determination of the iron content in SWCNT samples. Iron content determined by UV-Vis spectroscopy, SQUID, and TGA in purified SWCNT samples. These analyses were conducted following a cumulative 6-hour steam treatment, involving both direct purification (1 x 6 h) and multiple steam-HCl iterative purifications (2 x 3 h, 3 x 2 h, 6 x 1 h). An HCl wash was conducted after each steam treatment.

To assess the sensitivity of the UV-Vis spectroscopic assay for the determination of the Fe content, all samples exposed to 6 h of steam were analyzed using this technique. This includes a single 6 h steam treatment (1 x 6 h) and also those samples that were subjected to multiple shorter steam treatments, namely 6 times 1 h (6 x 1 h), 3 times 2 h (3 x 2 h) and 2 times 3 h (2 x 3 h). Samples for UV-Vis analyses were prepared by treating the collected residue after TGA with HCl. The obtained solution was then analyzed using UV-Vis spectroscopy. Table 1 and Fig 3 provide a summary of the iron content as determined by the different techniques employed in the present study.

**Table 1:** Amount of Fe determined by different techniques, and I_D_/I_G_ Raman ratio of SWCNT samples purified with steam for a total cumulative time of 6 h. This includes a single 6 h steam treatment (1 x 6 h) and also samples that were subjected to multiple shorter treatments, namely 6 times for 1 h (6 x 1 h), 3 times for 2 h (3 x 2 h) and 2 times for 3 h (2 x 3 h). SQUID values correspond to the 6 h points shown in Fig 1B).

Protocol (repetition x steam time)	TGA (Fe, wt. %)	SQUID (Fe, wt. %)	UV-Vis (Fe, wt. %)	I_D_/I_G_
**1 x 6 h**	1,62	0,51	0,64	0,11 ± 0,04
**2 x 3 h**	1,34	0,39	0,43	0,14 ± 0,04
**3 x 2 h**	1,28	0,28	0,39	0,14 ± 0,01
**6 x 1 h**	1,05	0,21	0,21	0,17 ± 0,07

As observed, the iron content determined by UV-Vis closely aligns with the data obtained from SQUID (Table 1, Fig 3). Nevertheless, the amount of Fe determined by TGA is notably higher, as previously discussed. This difference could be attributed to the presence of other inorganic compounds in the samples, such as alumina or silica, that could have been introduced during the synthesis or purification of SWCNTs. The presence of such impurities contributes to the total amount of inorganic residue, which is detected by TGA.

**Fig 4:**
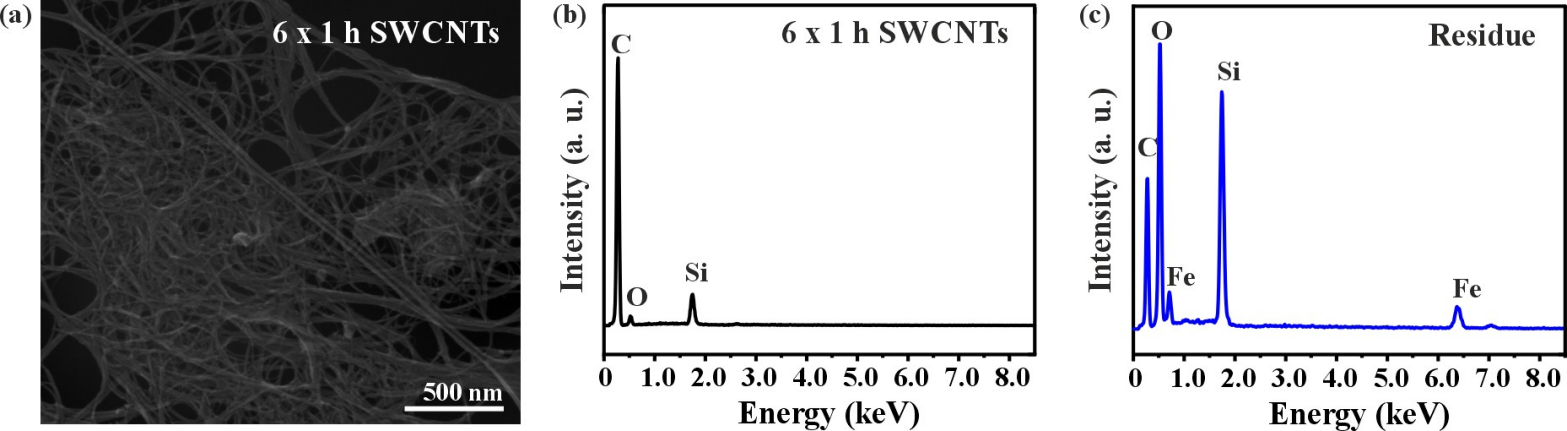
Determination of morphology and residual impurities in 6 x 1 h steam and HCl purified SWCNTs. a) SEM image of purified SWCNTs. b) EDX analysis of purified SWCNTs. c) EDX analysis of the solid residue collected after the oxidation of the purified SWCNTs.

Energy-dispersive X-ray spectroscopy (EDX) was next performed in the 6 x 1 h steam and HCl treated sample in a scanning electron microscope (SEM). Fig 4A shows a SEM image of purified SWCNTs (6 x 1 h) were several SWCNT bundles can be observed. EDX was performed in different areas of the sample, but due to the low amount of inorganic impurities it was difficult to assess the overall sample composition. An EDX analysis of the purified SWCNTs is shown in Fig 4B. In this specific area the presence of silica impurities can be detected, but this was not the case in other analyzed areas. To get a better overview of the sample composition, the 6 x 1 h treated sample was oxidized under oxygen to remove carbon and the resulting solid residue was analyzed by EDX (Fig 4C). As it can be seen in the analysis both Si and Fe appear to be present as impurities in the sample. The presence of oxygen is attributed to silica and to iron oxide, after the oxidation of the material. A carbon tape was employed as support. Silica and alumina have been previously observed as non-magnetic impurities in carbon nanotube samples, apart from graphitic particles and fullerenes [67], because these materials are widely employed during the growth and/or post-purification of the CNTs [52,66,68].

Even if silica was present during UV-Vis analysis, the quantitative assessment of iron using UV-Vis spectroscopy remains specifically associated with this element. The same principle applies to SQUID analysis, which exclusively detects magnetic materials and would be, therefore, insensitive to the presence of silica. Consequently, the UV-Vis methodology presented for the quantitative analysis of iron impurities in SWCNT samples, without the need of additional complexing agents, emerges as a straightforward, rapid, and cost-effective alternative to SQUID and TGA.

To complete the study, Raman spectroscopy was employed to examine all the samples that underwent a total of six hours of steam treatment (1 x 6 h, 2 x 3 h, 3 x 2 h, 6 x 1 h). Table 1 provides the calculated D to G band intensity ratios, widely employed to evaluate the degree of structural defects in SWCNTs. The data in the table demonstrates that this ratio remains essentially unchanged, with variations falling within statistical error.

The combination of the above analyses confirms that a multi-step treatment is more efficient for the removal of iron in SWCNT samples, than a single-step treatment with steam for the same total amount of time. Furthermore, it is worth stressing than the multi-step treatment does not result in an increase in the number of structural defects. As mentioned, purified CNTs are of interest for a variety of applications including sensors or their potential use in the biomedical field. CNTs have been advocated as promising materials for drug delivery, biomedical imaging and targeted therapies. The use of nanotubes, and nanoparticles in general, in the biomedical field offer a unique platform to adjust essential properties such as solubility, diffusivity, pharmacokinetic profile, blood-circulation time and toxicity. Therefore, several nanoformulations are being explored in a wide variety of biomedical applications [69–75].

When it comes the quantitative determination of the amount of metal in SWCNTs, the present study complements the early work by Agustina *et al*. [66] on multi-walled CNTs thus validating UV-Vis spectroscopy as a fast an convenient method for the assessment of catalytic particles in both as-received and purified carbon nanotube samples. A simplified method is used herein that does not require the addition of complexing agents and buffers to adjust the pH of the solution. It also does not require the digestion of the sample in solutions of strong acids to oxidize the graphitic particles surrounding the catalysts. It is worth noting that the remaining residue after TGA, under an oxidizing atmosphere, is enough for a precise determination of the metal content. We believe that the proposed strategy is highly versatile and could be employed to evaluate metal content in other types of nanomaterials.

## Conclusions

In conclusion, we have shown that a multi-step purification approach, involving iterative steam-HCl treatments, is more efficient in reducing the amount of catalyst in SWCNT samples compared to a single steam and HCl purification. Analysis of the samples reveals that this approach does not introduce structural defects.

We have also proposed a simplified UV-Vis protocol for the quantitative assessment of Fe in samples of SWCNTs that consists on the dissolution, in HCl, of the solid remaining upon the complete combustion of the sample, thus eliminating the need of additional complexing agents.

## References

[1] Maignan C, Alauzun JG, Flahaut E, Monconduit L, Boury B. Graphene oxide Composites: A versatile material used as protective layer, solid-state electrolyte, and gel electrolyte in metal batteries. Chem Eng J. 2024;485:149616.

[2] Singh V, Zhang J, Chen J, Salzmann CG, Tiwari MK. Precision Covalent Organic Frameworks for Surface Nucleation Control. Adv Mater. 2023 Sep 1;35(38):2302466. doi: 10.1002/adma.202302466 37311152

[3] Zinatloo-Ajabshir S, Baladi M, Salavati-Niasari M. Enhanced visible-light-driven photocatalytic performance for degradation of organic contaminants using PbWO_4_ nanostructure fabricated by a new, simple and green sonochemical approach. Ultrason Sonochem. 2021;72:105420. 33385636 10.1016/j.ultsonch.2020.105420 PMC7803816

[4] Quirós-Díez EP, Herreros-Lucas C, Vila-Fungueiriño JM, Vizcaíno-Anaya L, Sabater-Algarra Y, Giménez-López M del C. Boosting Oxygen Reduction Reaction Selectivity in Metal Nanoparticles with Polyoxometalates. Small Methods. 2024:2301805. doi: 10.1002/smtd.202301805 38517266 PMC11672175

[5] Ebrahimi A, Krivosudský L, Cherevan A, Eder D. Polyoxometalate-based porphyrinic metal-organic frameworks as heterogeneous catalysts. Coord Chem Rev. 2024;508:215764.

[6] Ferrer-Ugalde A, Sandoval S, Pulagam KR, Muñoz-Juan A, Laromaine A, Llop J, et al. Radiolabeled Cobaltabis(dicarbollide) Anion–Graphene Oxide Nanocomposites for In Vivo Bioimaging and Boron Delivery. ACS Appl Nano Mater. 2021 Feb 26;4(2):1613–25.

[7] Ashoub MH, Razavi R, Heydaryan K, Salavati-Niasari M, Amiri M. Targeting ferroptosis for leukemia therapy: exploring novel strategies from its mechanisms and role in leukemia based on nanotechnology. Eur J Med Res. 2024;29(1):224. doi: 10.1186/s40001-024-01822-7 38594732 PMC11003188

[8] Kadam SA, Sandoval S, Bastl Z, Simkovičová K, Kvítek L, Jašík J, et al. Cyclohexane Oxidative Dehydrogenation on Graphene-Oxide-Supported Cobalt Ferrite Nanohybrids: Effect of Dynamic Nature of Active Sites on Reaction Selectivity. ACS Catal. 2023 Oct 20;13(20):13484–505. doi: 10.1021/acscatal.3c02592 37881789 PMC10594591

[9] Alam RB, Ahmad MH, Islam MR. Improved electrochemical performance of multi-walled carbon nanotube reinforced gelatin biopolymer for transient energy storage applications. PLoS One. 2023;18(11):e0288113. doi: 10.1371/journal.pone.0288113 37943871 PMC10635558

[10] Bura C, Mocan T, Grapa C, Mocan L. Carbon Nanotubes-Based Assays for Cancer Detection and Screening. Pharmaceutics. 2022;14(4):781. doi: 10.3390/pharmaceutics14040781 35456615 PMC9028434

[11] Wang JT-W, Klippstein R, Martincic M, Pach E, Feldman R, Šefl M, et al. Neutron Activated ^153^Sm Sealed in Carbon Nanocapsules for in Vivo Imaging and Tumor Radiotherapy. ACS Nano. 2020;14(1):129. doi: 10.1021/acsnano.9b04898 31742990

[12] Adorinni S, Rozhin P, Marchesan S. Smart Hydrogels Meet Carbon Nanomaterials for New Frontiers in Medicine. Biomedicines. 2021;9(5):570. doi: 10.3390/biomedicines9050570 34070138 PMC8158376

[13] Gul S, Abdullah M, Zafar M, Ali I, Ullah Khan N, Younas M, et al. Development of hybrid composite by integrating functionalized multi-walled carbon nanotubes (f-MWCNTs) with glass fiber reinforced polyester composite. PLoS One. 2022 Dec 29;17(12):e0279647. doi: 10.1371/journal.pone.0279647 36580482 PMC9799322

[14] Chen T-Y, Vedhanarayanan B, Lin S-Y, Shao L-D, Sofer Z, Lin J-Y, et al. Electrodeposited NiSe on a forest of carbon nanotubes as a free-standing electrode for hybrid supercapacitors and overall water splitting. J Colloid Interface Sci. 2020;574:300–11. doi: 10.1016/j.jcis.2020.04.034 32335481

[15] Zhang W, Guillén-Soler M, Moreno-Da Silva S, López-Moreno A, González LR, Giménez-López M del C, et al. Mechanical interlocking of SWNTs with N-rich macrocycles for efficient ORR electrocatalysis. Chem Sci. 2022;13(33):9706–12. doi: 10.1039/d2sc02346f 36091908 PMC9400660

[16] Gonçalves G, Sandoval S, Llenas M, Ballesteros B, Da Ros T, Bortolussi S, et al. Lithium halide filled carbon nanocapsules: Paving the way towards lithium neutron capture therapy (LiNCT). Carbon N Y. 2023;208:148.

[17] Salavati-Niasari M, Badiei A, Saberyan K. Oxovanadium(IV) salophen complex covalently anchored to multi-wall carbon nanotubes (MWNTs) as heterogeneous catalyst for oxidation of cyclooctene. Chem Eng J. 2011;173(2):651–8.

[18] Heydariyan Z, Monsef R, Dawi EA, Salavati-Niasari M. EuMnO_3_/EuMn_2_O_5_/MWCNT nanocomposites: Insights into synthesis and application as potential materials for development of hydrogen storage capacity. Fuel. 2023;351:128885.

[19] Koyama S, Kim YA, Hayashi T, Takeuchi K, Fujii C, Kuroiwa N, et al. In vivo immunological toxicity in mice of carbon nanotubes with impurities. Carbon N Y. 2009;47(5):1365–72.

[20] Sharma HS, Hussain S, Schlager J, Ali SF, Sharma A. Influence of Nanoparticles on Blood–Brain Barrier Permeability and Brain Edema Formation in Rats BT—Brain Edema XIV. In: Czernicki Z, Baethmann A, Ito U, Katayama Y, Kuroiwa T, Mendelow D, editors. Vienna: Springer Vienna; 2010. p. 359–64. 10.1007/978-3-211-98811-4_65 19812977

[21] Stuart E, Pumera M. Impurities within carbon nanotubes govern the electrochemical oxidation of substituted hydrazines. Phys Chem Chem Phys. 2011 Jun 14;13:10818–22. doi: 10.1039/c1cp20176j 21556440

[22] Banks CE, Crossley A, Salter C, Wilkins SJ, Compton RG. Carbon Nanotubes Contain Metal Impurities Which Are Responsible for the “Electrocatalysis” Seen at Some Nanotube-Modified Electrodes. Angew Chemie Int Ed. 2006 Apr 10;45(16):2533–7. doi: 10.1002/anie.200600033 16544355

[23] Pumera M, Miyahara Y. What amount of metallic impurities in carbon nanotubes is small enough not to dominate their redox properties? Nanoscale. 2009 Nov 1;1:260–5. doi: 10.1039/b9nr00071b 20644847

[24] Batchelor-McAuley C, Wildgoose GG, Compton RG, Shao L, Green MLH. Copper oxide nanoparticle impurities are responsible for the electroanalytical detection of glucose seen using multiwalled carbon nanotubes. Sensors Actuators B Chem. 2008;132(1):356–60.

[25] Bussy C, Bianco A, Prato M, Kostarelos K. Primary microglia maintain their capacity to function despite internalisation and intracellular loading with carbon nanotubes. Nanoscale Horizons. 2017;2(5):284–96. doi: 10.1039/c7nh00055c 32260684

[26] Li D, Al-Jamal KT. siRNA Design and Delivery Based on Carbon Nanotubes BT—Design and Delivery of SiRNA Therapeutics. In: Ditzel HJ, Tuttolomondo M, Kauppinen S, editors. New York, NY: Springer US; 2021. p. 181–93. 10.1007/978-1-0716-1298-9_12 33928577

[27] Soligo M, Felsani FM, Da Ros T, Bosi S, Pellizzoni E, Bruni S, et al. Distribution in the brain and possible neuroprotective effects of intranasally delivered multi-walled carbon nanotubes. Nanoscale Adv. 2021;3(2):418–31. doi: 10.1039/d0na00869a 36131737 PMC9418508

[28] Alegret N, Dominguez-Alfaro A, González-Domínguez JM, Arnaiz B, Cossío U, Bosi S, et al. Three-Dimensional Conductive Scaffolds as Neural Prostheses Based on Carbon Nanotubes and Polypyrrole. ACS Appl Mater Interfaces. 2018 Dec 19;10(50):43904–14. doi: 10.1021/acsami.8b16462 30475577

[29] Ribeiro H, Schnitzler MC, da Silva WM, Santos AP. Purification of carbon nanotubes produced by the electric arc-discharge method. Surfaces and Interfaces. 2021;26:101389.

[30] Desforges A, Bridi AV, Kadok J, Flahaut E, Le Normand F, Gleize J, et al. Dramatic enhancement of double-walled carbon nanotube quality through a one-pot tunable purification method. Carbon N Y. 2016;110:292–303.

[31] Ghasemzadeh S, Hosseini-Monfared H, Ghorbanloo M, Beglau THY, Rademacher L, Spieß A, et al. Scalable synthesis of SWCNT via CH_4_/N_2_ gas: The effects of purification on photocatalytic properties of CNT/TiO2 nanocomposite. J Environ Chem Eng. 2022;10(5):108440.

[32] Khanbolouki P, Tehrani M. Purification, structural evolutions, and electrical properties of carbon nanotube yarns processed via incandescent annealing. Carbon N Y. 2020;168:710–8.

[33] Romanov SA, Alekseeva AA, Khabushev EM, Krasnikov D V, Nasibulin AG. Rapid, efficient, and non-destructive purification of single-walled carbon nanotube films from metallic impurities by Joule heating. Carbon N Y. 2020;168:193–200.

[34] Lin Y, Kim J-W, Connell JW, Lebrón-Colón M, Siochi EJ. Purification of Carbon Nanotube Sheets. Adv Eng Mater. 2015 May 1;17(5):674–88.

[35] Podlesny B, Olszewska B, Yaari Z, Jena P V, Ghahramani G, Feiner R, et al. En route to single-step, two-phase purification of carbon nanotubes facilitated by high-throughput spectroscopy. Sci Rep. 2021;11(1):10618. doi: 10.1038/s41598-021-89839-4 34011997 PMC8134628

[36] Galassi T V, Antman-Passig M, Yaari Z, Jessurun J, Schwartz RE, Heller DA. Long-term in vivo biocompatibility of single-walled carbon nanotubes. PLoS One. 2020 May 6;15(5):e0226791. doi: 10.1371/journal.pone.0226791 32374764 PMC7202660

[37] Gomez V, Irusta S, Lawal OB, Adams W, Hauge RH, Dunnill CW, et al. Enhanced purification of carbon nanotubes by microwave and chlorine cleaning procedures. RSC Adv. 2016;6(14):11895–902.

[38] Qiao Z, Zhao L, Li N, Zhang J, Zhao K, Ji D, et al. Highly efficient and environmental-friendly separation and purification of carbon nanotubes from molten salt via ultrasound-assisted carbonation. Sep Purif Technol. 2023;306:122630.

[39] Yang X, Yang M, Zhang H, Zhao J, Zhang X, Li Q. Electro-purification of carbon nanotube networks without damaging the assembly structure and crystallinity. Appl Surf Sci. 2018;442:232–8.

[40] Gurova OA, Arhipov VE, Koroteev VO, Guselnikova TY, Asanov IP, Sedelnikova O V, et al. Purification of Single-Walled Carbon Nanotubes Using Acid Treatment and Magnetic Separation. Phys status solidi. 2019 Sep 1;256(9):1800742.

[41] Andrews R, Jacques D, Qian D, Dickey EC. Purification and structural annealing of multiwalled carbon nanotubes at graphitization temperatures. Carbon N Y. 2001;39(11):1681–7.

[42] Méténier K, Bonnamy S, Béguin F, Journet C, Bernier P, Lamy de La Chapelle M, et al. Coalescence of single-walled carbon nanotubes and formation of multi-walled carbon nanotubes under high-temperature treatments. Carbon N Y. 2002;40(10):1765–73.

[43] Yudasaka M, Ichihashi T, Kasuya D, Kataura H, Iijima S. Structure changes of single-wall carbon nanotubes and single-wall carbon nanohorns caused by heat treatment. Carbon N Y. 2003;41(6):1273–80.

[44] Im Y-O, Lee S-H, Kim T, Park J, Lee J, Lee K-H. Utilization of carboxylic functional groups generated during purification of carbon nanotube fiber for its strength improvement. Appl Surf Sci. 2017;392:342–9.

[45] Battigelli A, Ménard-Moyon C, Da Ros T, Prato M, Bianco A. Endowing carbon nanotubes with biological and biomedical properties by chemical modifications. Adv Drug Deliv Rev. 2013;65(15):1899–920. doi: 10.1016/j.addr.2013.07.006 23856410

[46] Eder D. Carbon nanotube-inorganic hybrids. Chem Rev. 2010;110(3):1348–85. doi: 10.1021/cr800433k 20108978

[47] Lin C-C, Chu BTT, Tobias G, Sahakalkan S, Roth S, Green MLH, et al. Electron transport behavior of individual zinc oxide coated single-walled carbon nanotubes. Nanotechnology. 2009;20(10):105703. doi: 10.1088/0957-4484/20/10/105703 19417531

[48] Dörling B, Sandoval S, Kankla P, Fuertes A, Tobias G, Campoy-Quiles M. Exploring different doping mechanisms in thermoelectric polymer/carbon nanotube composites. Synth Met. 2017;225:70.

[49] Tanaka H, Goto T, Hamada K, Ohashi K, Osawa T, Sugime H, et al. Safe and damage-less dry-purification of carbon nanotubes using FeCl3 vapor. Carbon N Y. 2023;212:118171.

[50] Ballesteros B, Tobias G, Shao L, Pellicer E, Nogués J, Mendoza E, et al. Steam purification for the removal of graphitic shells coating catalytic particles and the shortening of single-walled carbon nanotubes. Small. 2008;4(9):1501. doi: 10.1002/smll.200701283 18702121

[51] King SG, McCafferty L, Stolojan V, Silva SRP. Highly aligned arrays of super resilient carbon nanotubes by steam purification. Carbon N Y. 2015;84:130–7.

[52] Cabana L, Ke X, Kepić D, Oro-Solé J, Tobías-Rossell E, Van Tendeloo G, et al. The role of steam treatment on the structure, purity and length distribution of multi-walled carbon nanotubes. Carbon N Y. 2015;93:1059.

[53] Sandoval S, Kepić D, Pérez Del Pino Á, György E, Gómez A, Pfannmoeller M, et al. Selective Laser-Assisted Synthesis of Tubular van der Waals Heterostructures of Single-Layered PbI2 within Carbon Nanotubes Exhibiting Carrier Photogeneration. ACS Nano. 2018;12(7):6648–56. doi: 10.1021/acsnano.8b01638 29975504

[54] Kierkowicz M, González-Domínguez JM, Pach E, Sandoval S, Ballesteros B, Da Ros T, et al. Filling Single-Walled Carbon Nanotubes with Lutetium Chloride: A Sustainable Production of Nanocapsules Free of Nonencapsulated Material. ACS Sustain Chem Eng. 2017 Mar;5(3):2501–8.

[55] Sandoval S, Tobias G, Flahaut E. Structure of inorganic nanocrystals confined within carbon nanotubes. Inorg Chim Acta. 2019;492:66.

[56] Martincic M, Vranic S, Pach E, Sandoval S, Ballesteros B, Kostarelos K, et al. Non-cytotoxic carbon nanocapsules synthesized via one-pot filling and end-closing of multi-walled carbon nanotubes. Carbon N Y. 2019;141:782–93.

[57] Kierkowicz M, Pach E, Fraile J, Domingo C, Ballesteros B, Tobias G. The Role of Temperature on the Degree of End-Closing and Filling of Single-Walled Carbon Nanotubes. Nanomaterials. 2021;11(12):3365. doi: 10.3390/nano11123365 34947714 PMC8704686

[58] Sandoval S, Tobias G. Encapsulation of Fullerenes: A Versatile Approach for the Confinement and Release of Materials Within Open-Ended Multiwalled Carbon Nanotubes. Front Bioeng Biotechnol. 2021;9:644793. doi: 10.3389/fbioe.2021.644793 33777916 PMC7987908

[59] Shao L, Tobias G, Huh Y, Green MLH. Reversible filling of single walled carbon nanotubes opened by alkali hydroxides. Carbon N Y. 2006;44(13):2855–8.

[60] Kashtiban RJ, Patrick CE, Ramasse Q, Walton RI, Sloan J. Picoperovskites: The Smallest Conceivable Isolated Halide Perovskite Structures Formed within Carbon Nanotubes. Adv Mater. 2023 Mar 1;35(10):2208575. doi: 10.1002/adma.202208575 36528852

[61] Hart M, Chen J, Michaelides A, Sella A, Shaffer MSP, Salzmann CG. One-Dimensional Pnictogen Allotropes inside Single-Wall Carbon Nanotubes. Inorg Chem. 2019 Nov 18;58(22):15216–24. doi: 10.1021/acs.inorgchem.9b02190 31693345

[62] Kashtiban RJ, Burdanova MG, Vasylenko A, Wynn J, Medeiros PVC, Ramasse Q, et al. Linear and Helical Cesium Iodide Atomic Chains in Ultranarrow Single-Walled Carbon Nanotubes: Impact on Optical Properties. ACS Nano. 2021 Aug 24;15(8):13389–98. doi: 10.1021/acsnano.1c03705 34370946

[63] Martinati M, Wenseleers W, Shi L, Pratik SM, Rohringer P, Cui W, et al. Electronic structure of confined carbyne from joint wavelength-dependent resonant Raman spectroscopy and density functional theory investigations. Carbon N Y. 2022;189:276–83.

[64] Kharlamova M V, Kramberger C. Phemenology of Filling, Investigation of Growth Kinetics and Electronic Properties for Applications of Filled Single-Walled Carbon Nanotubes. Nanomaterials. 2023;13(2):314. doi: 10.3390/nano13020314 36678067 PMC9862314

[65] Santidrián A, Kierkowicz M, Pach E, Darvasiová D, Ballesteros B, Tobias G, et al. Charge transfer in steam purified arc discharge single walled carbon nanotubes filled with lutetium halides. Phys Chem Chem Phys. 2020;22(18):10063–75. doi: 10.1039/d0cp01408g 32338257

[66] Agustina E, Goak J, Lee S, Seo Y, Park J-Y, Lee N. Simple and Precise Quantification of Iron Catalyst Content in Carbon Nanotubes Using UV/Visible Spectroscopy. ChemistryOpen. 2015 Oct 1;4(5):613–9. doi: 10.1002/open.201500096 26491641 PMC4608529

[67] Ambrosi A, Pumera M. Nanographite Impurities Dominate Electrochemistry of Carbon Nanotubes. Chem–A Eur J. 2010 Sep 24;16(36):10946–9. doi: 10.1002/chem.201001584 20715215

[68] Kolodiazhnyi T, Pumera M. Towards an Ultrasensitive Method for the Determination of Metal Impurities in Carbon Nanotubes. Small. 2008 Sep 1;4(9):1476–84. doi: 10.1002/smll.200800125 18680097

[69] Mahata MK, De R, Lee KT. Near-Infrared-Triggered Upconverting Nanoparticles for Biomedicine Applications. Biomedicines. 2021;9(7):756. doi: 10.3390/biomedicines9070756 34210059 PMC8301434

[70] Sturm L, Schwemberger B, Menzel U, Häckel S, Albers CE, Plank C, et al. In Vitro Evaluation of a Nanoparticle-Based mRNA Delivery System for Cells in the Joint. Biomedicines. 2021;9(7):794. doi: 10.3390/biomedicines9070794 34356857 PMC8301349

[71] Akhmedov S, Afanasyev S, Trusova M, Postnikov P, Rogovskaya Y, Grakova E, et al. Chemically Modified Biomimetic Carbon-Coated Iron Nanoparticles for Stent Coatings: In Vitro Cytocompatibility and In Vivo Structural Changes in Human Atherosclerotic Plaques. Biomedicines. 2021;9(7):802. doi: 10.3390/biomedicines9070802 34356866 PMC8301308

[72] Oliveira FA, Nucci MP, Mamani JB, Alves AH, Rego GNA, Kondo AT, et al. Multimodal Tracking of Hematopoietic Stem Cells from Young and Old Mice Labeled with Magnetic–Fluorescent Nanoparticles and Their Grafting by Bioluminescence in a Bone Marrow Transplant Model. Biomedicines. 2021;9(7):752. doi: 10.3390/biomedicines9070752 34209598 PMC8301491

[73] Florensa M, Llenas M, Medina-Gutiérrez E, Sandoval S, Tobías-Rossell G. Key Parameters for the Rational Design, Synthesis, and Functionalization of Biocompatible Mesoporous Silica Nanoparticles. Pharmaceutics. 2022;14(12):2703. doi: 10.3390/pharmaceutics14122703 36559195 PMC9788600

[74] Pelin M, Lin H, Gazzi A, Sosa S, Ponti C, Ortega A, et al. Partial Reversibility of the Cytotoxic Effect Induced by Graphene-Based Materials in Skin Keratinocytes. Nanomaterials. 2020;10(8):1602. doi: 10.3390/nano10081602 32824126 PMC7466707

[75] Malanagahalli S, Murera D, Martín C, Lin H, Wadier N, Dumortier H, et al. Few Layer Graphene Does Not Affect Cellular Homeostasis of Mouse Macrophages. Nanomaterials. 2020;10(2):228. doi: 10.3390/nano10020228 32013038 PMC7074970

