# Supplementary material for: UV-Vis quantification of the iron content in iteratively steam and HCl purified single-walled carbon nanotubes
Source: PLoS One. 2024 May 10;19(5):e0303359. doi: 10.1371/journal.pone.0303359 (PMC11086872; doi:10.1371/journal.pone.0303359)
Supplement: Attachment — Submitted filename: Response to Reviewers-R2.pdf [file pone.0303359.s002.pdf]

We would like to thank the reviewer for the additional suggestions to improve the quality of the manuscript. The manuscript has been modified to include the requested changes. Yellow highlighting has been employed for ease of comparison with the initially submitted version.

Reviewer #1: Manuscript ID: PONE-D-23-37335R1

I will consider publishing your paper entitled "UV-Vis quantification of the iron content in iteratively steam and HCl purified singlewalled carbon nanotubes"; authors present a multi-step purification treatment that combines the use of steam and hydrochloric acid in an iterative manner. After reading authors' responses, I feel that my initial concerns have been partially addressed, but there are still some major concepts I cannot agree with.

Comments:

For Responses 1 and 2, I am grateful for additional information for the backgrounds.

We are glad that the concerns were properly addressed.

For Response 3, it is suggested that this paper can provide more lists. The structure of the manuscript might need a major adjustment for a better understanding. To make more convincing, in the discussions, the authors should be considered some publication on the improvement of the explanation such as:

- Fuel, 351 (2023) 128885.

- Journal of Materials Research and Technology, 23 (2023) 3126-3136.

- Applied Surface Science, 255 (2009) 7610-7617.

- Chemical Engineering Journal, 173 (2011) 651-658.

- Separation and Purification Technology, 185 (2017) 140-148.

Journal of Molecular Liquids 242 (2017) 447-455; Journal of alloys and compounds 617 (2014) 627-632; Diamond and Related Materials 79 (2017) 133-144;

Following the suggestion of the reviewer we have modified the introduction by including an initial sentence to proceed a broader perspective. Several of the suggested references have been added either at the end of this sentence or when discussing the potential applications of carbon nanotubes.

For Responses 4-8, I am grateful for additional information for the backgrounds.

We are glad that the concerns were properly addressed.

For Response 9, in "Result and Discussions" section, the authors explained irrelevant and unnecessary subjects. For the characterizations, the results must be compared with other studies. You are requested to add some to amplify how this research work contributes to forwarding the field of study.

To fully address the concerns raised by the reviewer and taking into account that in the first report the reviewer mentioned in point 9 "It is suggested to evaluate the chemical composition of fabricated sample by other methods such as elemental mapping, to get clear evidence to readers for formation samples without impurities.", we have performed additional experiments (EDX & SEM) and also

improved the discussion on this topic. A new figure (Fig 4) has been included and the following paragraph has been added: *"Energy-dispersive X-ray spectroscopy (EDX) was next performed in the 6 x 1 h steam and HCl treated sample in a scanning electron microscope (SEM). Fig 4A shows a SEM image of purified SWCNTs (6 x 1 h) were several SWCNT bundles can be observed. EDX was performed in different areas of the sample, but due to the low amount of inorganic impurities it was difficult to assess the overall sample composition. An EDX analysis of the purified SWCNTs is shown in Fig 4B. In this specific area the presence of silica impurities can be detected, but this was not the case in other analyzed areas. To get a better overview of the sample composition, the 6 x 1 h treated sample was oxidized under oxygen to remove carbon and the resulting solid residue was analyzed by EDX (Fig 4C). As it can be seen in the analysis both Si and Fe appear to be present as impurities in the sample. The presence of oxygen is attributed to silica and to iron oxide, after the oxidation of the material. A carbon tape was employed as support. Silica and alumina have been previously observed as non-magnetic impurities in carbon nanotube samples, apart from graphitic particles and fullerenes (67), because these materials are widely employed during the growth and/or post-purification of the CNTs (52,66,68)."*

For Response 10, I am grateful for additional information for the backgrounds.

We are glad that the concerns were properly addressed.
