# Supplementary material for: UV-Vis quantification of the iron content in iteratively steam and HCl purified single-walled carbon nanotubes
Source: PLoS One. 2024 May 10;19(5):e0303359. doi: 10.1371/journal.pone.0303359 (PMC11086872; doi:10.1371/journal.pone.0303359)
Supplement: Attachment — Submitted filename: Response to Reviewers.docx [file pone.0303359.s001.docx]

We would like to thank the reviewer for the positive comments and suggestions to improve the quality of the manuscript. The manuscript has been modified to include the requested changes. “Track changes” have been employed for ease of comparison with the initially submitted version.

**Reviewer #1: Manuscript ID: PONE-D-23-37335**

**UV-Vis quantification of the metal content in iteratively purified single-walled carbon nanotubes**

**The manuscript entitled "UV-Vis quantification of the metal content in iteratively purified single-walled carbon nanotubes" was reviewed. This paper present a multi-step purification treatment that combines the alternated use of steam and hydrochloric acid. This manuscript can be accepted for publishing “PLOS ONE” but I have some major remarks before it can be publishable.**

**1. The abstract is unattractive. It is suggested that the importance of this research work be written in detail.**

Following the suggestion of the reviewer the abstract has been revised highlighting the importance of the research work.

**2. The title is informative and relevant, it could be more specific.**

A more specific title is now provided replacing “metal” for “iron” and detailing the type of purification “steam and HCl”.

**3. Authors have claimed “Carbon nanotubes (CNTs) can be employed in a wide variety of applications that range from nanoelectronics, sensors, catalysis, composite materials and cancer therapy to name some.”, I have read and evaluated the manuscript and in my opinion the submission does not yet sufficiently justify publication. The whole generalization for this paper should be given in the introduction. Discuss the shortcomings of previous work and the gaps and how this work intends to fill those gaps. Related references should be cited:**

**Materials Research Bulletin 48 (4) (2013) 1660-1667; Ultrasonics Sonochemistry 72 (2021), 105420; international journal of hydrogen energy 42 (39) (2017) 24846-24860; Polyhedron 28 (14) (2009) 3005-3009; Journal of Molecular Liquids 242 (2017) 447-455; Journal of alloys and compounds 617 (2014) 627-632; Diamond and Related Materials 79 (2017) 133-144**

The introduction has been fully revised following the advice of the reviewer. We have also increased the number of references related to the work as suggested by the reviewer. Nevertheless, we have not included the references provided by the reviewer because they do not deal with carbon nanotubes, as it can be seen in their titles:

**Materials Research Bulletin 48 (4) (2013) 1660-1667 -** Preparation of TiO2 nanoparticles by using tripodal tetraamine ligands as complexing agent via two-step sol–gel method and their application in dye-sensitized solar cells.

**Ultrasonics Sonochemistry 72 (2021), 105420 -** Enhanced visible-light-driven photocatalytic performance for degradation of organic contaminants using PbWO4 nanostructure fabricated by a new, simple and green sonochemical approach.

**international journal of hydrogen energy 42 (39) (2017) 24846-24860 -** Removal of malachite green (a toxic dye) from water by cobalt ferrite silica magnetic nanocomposite: Herbal and green sol-gel autocombustion synthesis

**Polyhedron 28 (14) (2009) 3005-3009 -** Pure cubic ZrO2 nanoparticles by thermolysis of a new precursor

**Journal of Molecular Liquids 242 (2017) 447-455 -** Synthesis of graphene quantum dots from corn powder and their application in reduce charge recombination and increase free charge carriers

**Journal of alloys and compounds 617 (2014) 627-632 -** Facile microwave synthesis, characterization, and solar cell application of selenium nanoparticles

**Diamond and Related Materials 79 (2017) 133-144 -** Facile reduction of graphene using urea in solid phase and surface modification by N-doped graphene quantum dots for adsorption of organic dyes

**4. In the Abstract, the authors should emphasize what results the characterizations indicate.**

Following the advice of the reviewer the Abstract has been modified stressing the results of the characterization.

**5. The idea of the research seems to be interesting but the set goals are not achieved. What the main significance of paper in comparison is of relates published works? What is the innovation of this article? Please highlight it in the article.**

As indicated above, the introduction has been revised to compare this study with other works and better explain the innovation of this article.

**6. Explain about the effective parameters which increase the yield of reactions?**

A discussion about the parameters that might increase the yield of reaction has also been added in page 9 of the manuscript. It reads as follows: *“The more iterations of steam + HCl are performed, using shorter steam treatment times, the lower becomes the amount of Fe in the samples. The reason behind this observation would need further investigation, but it could be due to a higher accessibility of steam to the whole SWCNT sample in the multi-step processing, because the material gets mixed during the acid treatment. Towards this end it would be interesting to perform the steam purification in a rotary tube furnace. Another possibility would be that a certain degree of reaction occurs between steam and SWCNT samples during heating and cooling of the furnace. This would result in slightly longer exposure times of the sample with steam when the purification is fractioned.”*

**7. Written is very week. In its current state, the level of English throughout the manuscript needs language polishing. Please check the manuscript and refine the language carefully.**

English language has been improved through the whole manuscript as it can be appreciated in the “track changes” version.

**8. Error bars in figures must be added.**

We appreciate the comment by the reviewer and agree that it would be better to add error bars in figures. Unfortunately, in this study error bars could not be included because only one sample was available for each data point. In fact, as already mentioned in the manuscript, we had to use two independent batches of as-received SWCNTs were employed for the experiments, which are no longer available. A new batch cannot be employed because the amount of impurities in as-received SWCNTs is always slightly different. Therefore, the resulting analysis could not be included in the already available figures. The importance of this work resides in the observed trends. We are confident that these are correct because steam and HCl have been widely employed before for different types and sources of CNTs and clear trends, using only a single data point, have been always obtained in all the studies. See for instance refs. 40 and 42.

**9. It is suggested to evaluate the chemical composition of fabricated sample by other methods such as elemental mapping, to get clear evidence to readers for formation samples without impurities.**

As mentioned the use of steam followed by HCl is widely documented and the chemical composition of the samples has been previously investigated. Reference to this previous studies has been included in the manuscript. The novelty of the work resides in the multistep steam + HCl treatment which will not modify the nature of the chemical composition of the sample.

**10. The Conclusions section is too long. It should be kept short and must be fully supported by the results reported.**

Following the suggestion of the reviewer the conclusion section has been greatly reduced.

**I recommend this work for publication after above mentioned revisions.**

We believe that we have fully addressed and properly answered to all the concerns raised by the reviewer, but please let us know if you feel that any further changes are needed.
